# Syphilis Communicated by a Kiss

**Published:** 1868-09

**Authors:** 


### SELECTED ARTICLES.

### ARTICLE IX.

### *Syphilis Communicated by a Kiss.*

Dr. A. M. Sigmund, of Shimersville Pa., reports the following cases in the *Humbolt Medical Archives*, of syphilis communicated by a kiss. Such instances are by no means unparalelled, but they are not, as he imagines, cases of communication of *tertiary* syphilis, properly so called. It should rather read *constitutional* syphilis. No doubt ought now to exist as to the communicability of syphilis from its later lesions.

On August 24th, 1867, I was called to see Miss M. H., aged 17 years, well developed, and to all appearance in good health, with the exception of a large and painful ulcer on her upper lip. Upon examination I found the ulcer to present the following characters: It was situated on the centre of the lip, extending from its anterior margin to near the frænum, oval in form, and, the lip being much swollen, about five-eights of an inch in its longest diameter. Its surface was hollow, as if scooped out, and covered with a layer of dirty greyish lymph; the edges were hard, slightly elevated, and sloping a little from within outward; the base well defined, and very hard, feeling, when pressed between thumb and finger, like a button or ring of fibro-cartilage.

On inquiry, I learned that about two weeks previous the lip had become indurated at the centre, where she had had a slight chap, after which the induration increased until it had involved the whole thickness of the lip, and then—about a week after—it commenced to ulcerate. On my informing her that the ulcer was undoubtedly of a syphilitic character, and asking her as to how and when she became infected, I received the following statement, which, from what I since have learned, I believe to be true. About three weeks before she had been at a picnic, and was there in company with a young man, (whom *I knew* to have been laboring under *tertiary syphilis*, and had also at that time some indolent sores on the inside of his lips,) and that at one time during the day, when he had been smoking a segar through a very beautiful amber mouth-piece, she playfully took it from him and placed it in her mouth. In the evening he accompanied her home, and in parting, impressed several kisses upon her lips, one of which was rather prolonged, in order as he said, to take a good parting kiss, as he would leave the neighborhood in a few days. She felt nothing unusual about the lip until about a week afterward, when the induration commenced; but thinks she had a slight chap or abrasion on it at that time, where the induration afterward occurred.

On further examination I found that no visible secondary or constitutional symptoms had as yet been developed, *and also, that there was no local disease, or evidence that there*

*had been any, on any other part of her body.* To all appearances, the disorder was as yet only local.

I applied solid nitrate of silver—the stick being brought to a fine point—thoroughly to every part of the ulcer, and the slough came away in a few days, the ulcer presenting rather an aggravated condition, when I re-applied the nitrate. From this time it commenced to heal—the nitrate being applied as often as was deemed necessary—and in about three weeks the sore was entirely healed, but considerable hardness remained for some time after, which, however, disappeared entirely under the subsequent constitutional treatment.

I remarked that when I first saw the patient, there was only the primary sore on the lips. In the course, however, of a few weeks—about five weeks from the appearance of the local lesion—secondary symptoms manifested themselves; such as the characteristic eruption, sore throat, pain in the ears, joints, etc., and enlargement of the cervical glands, with pain extending to the mastoid processes on both sides. She suffered also for some time from rheumatism (syphilitic) of the left arm, preventing its use; she also had some non-suppurating buboes, but only on the left side. The eruption as I have said, was markedly characteristic, and was most numerous on the forehead, scalp, face, neck, breast and arms; there was some also on the body and lower extremities, but not so numerous. It was at its height about ten days from its appearance, remained stationary about a week, and had disappeared again in three weeks more. The sore throat—not ulcerated, but only a little inflamed—enlargement of the glands, pain in the ears, and some soreness of the joints remained some weeks, longer. The whole course of treatment lasted between three and four months, when I discharged her seemingly cured. I have seen her but a few days since, and she tells me that she continues in perfect health.

The treatment, constitutionally, consisted of iodide of potassium, in doses ranging from five to fifteen grains, three times a day, given in compound syrup of sarsaparilla, with the bi-chloride of mercury in half-grain doses until the gums were slightly touched, when it was omitted; morphine was given to relieve the pain.

A few days after I was called to see this case, a young man came to my office, stating that he had a sore lip, which pained him considerably, and that he felt very uneasy about it, "especially as he had seen a lady having a very sore lip." On asking him more particularly, I learned that he had been in company with this same Miss M. H. a few days after the sore on her lip commenced, and not thinking of any danger, had kissed her. The sore was not as yet large, but had the characteristic appearance of indurated chancre. I applied nitrate of silver thoroughly then, and again in a few days after, after which it healed nicely, and then gave the usual constitutional treatment, and up to this time no constitutional symptoms have appeared

These cases are interesting as instances of the disease being communicated in rather an unusual channel.

The first case is also particularly interesting, as tending to determine the mooted point as to the comunicabillity of tertiary syphilis. There can be no question that the young man had at the time well defined tertiary syphilis; the sore on the young lady's lip was a "well-marked initial lesion," and followed by "characteristic constitutional syphilis.

---

